# Across-surface distances after one- and two-stage palatoplasty in children with oral cleft

**DOI:** 10.1590/1414-431X2024e13805

**Published:** 2024-10-14

**Authors:** E.C.P. Ambrosio, M.T.O.P. Bergamo, C. Sforza, C.F.C. Carrara, M.A.A.M. Machado, T.M. Oliveira

**Affiliations:** 1 Hospital de Reabilitação de Anomalias Craniofaciais, Universidade de São Paulo, Bauru, SP, Brasil; 2 Department of Cariology, Restorative Sciences, Endodontics, School of Dentistry, University of Michigan, Ann Arbor, MI, USA; 3 Department of Biomedical Sciences for Health, Functional Anatomy Research Center, Faculty of Medicine and Surgery, University of Milan, Milan, MI, Italy; 4 Departamento de Odontopediatria, Ortodontia e Sáude Coletiva, Faculdade de Odontologia de Bauru, Universidade de São Paulo, Bauru, SP, Brasil

**Keywords:** Cleft lip, Cleft palate, Infant, Palate, Imaging, Three-dimensional

## Abstract

The goal of this study was to digitally evaluate the development of maxillary dental arches of children with unilateral cleft lip and palate treated with one- and two-stage palatal closure. One hundred and sixty-eight digitized dental models of cheiloplasty and one-stage palatoplasty (G1) and cheiloplasty and two-stage palatoplasty (G2) were evaluated at preoperative time 1 (T1), preoperative time 2 (T2), and postoperative (T3). The following surface distances were evaluated: across surface distance; cleft widths anterior (P-P′) and posterior (U-U′) cleft widths, intercanine width (C-C′), and intertuberosity width (T-T′); smallest (P′-T′) and largest (P-T) segment lengths; and smallest (C′-D′) and largest (C-D) segment cleft depths. In G1, P-P′, U-U′, and C-C′ reduced at T2, unlike P′-T′ (P<0.05). P-T and C′-D′ distances increased at T3 (P<0.05), while C-D increased at all stages (P<0.001). In G2, U-U′ and C-C′ reduced at T2 (P<0.05), while P′-T′, P-T, C′-D′, and C-D′ increased at T3 (P<0.001). In an intergroup analysis of growth rate, G2 showed higher growth percentages compared to G1, in which C′-D′ was significant (P=0.038). Furthermore, C′-D′ presented a coefficient of determination of 0.076 (P=0.039). In conclusion, dental arch development is influenced by the rehabilitation protocol. However, in the sample evaluated, the comparison suggested that individuals whose palate was operated on in two stages had the most favorable palatal growth.

## Introduction

Usually, children with cleft lip and palate begin the multi-professional rehabilitation protocol a few months after birth (1). The aim is always to significantly improve the functional and esthetic aspects and the social well-being of the individual and their family (1). Although the stages and surgical techniques of the rehabilitation protocol vary, every individual undergoes plastic surgeries to repair the lip (cheiloplasty) (2-4) and palate (palatoplasty) (5-7), as they are fundamental steps in rehabilitation (8).

Several surgical techniques, such as Tennison, Randall, and Millard, have been proposed for cheiloplasty. The Tennison method involves a triangular flap that preserves the cupid's bow (2). In the Randall method, a triangular flap is also made, and the scar remains in the philtral columns (3), while the Millard technique involves advancing and rotating the flap for lip reconstruction (4). To this day, the latter is widely used by rehabilitation centers. Palatoplasty is a surgical procedure performed in one or two stages (8). The Hans Pichler, Sommerlad, and von Langenbeck techniques stand out. The Hans Pichler method repairs the hard palate (5), while the Sommerlad repairs the soft palate (6). The von Langenbeck technique involves anterior and posterior pediculate mucoperiosteal flaps to close the oral mucosa and detachment and synthesis of the nasal mucosa (7).

Rehabilitation protocols should be continuously monitored to verify whether the proposed results are achieved (1). Through a series of parameters, such as analysis of radiographic images, three-dimensional digitized dental arches, patient interviews, chart reviews, and clinical and scientific discussions, it is possible to verify factors that can be modified or maintained, always with the aim of achieving the patient's well-being (9-11). Digital analysis of dental arches has been a frequently used approach (12-14), and among its various advantages, the absence of ionizing radiation in image capture stands out. In addition, this type of analysis, done through a software, allows diverse anthropometric evaluations (such as area, volume, angle, and superimposition) (15). While the straight-line method has been widely applied in the analyses (12-14), the opposite is true for the across-surface distance method. Thus, the present study applied the across-surface distance analyses and evaluated the rehabilitation protocol longitudinally.

The goal of this study was to digitally evaluate the development of maxillary dental arches of children with unilateral cleft lip and palate treated with one- and two-stage palatal closure.

## Material and Methods

This retrospective study was conducted following the ethical principles of the Declaration of Helsinki. It was reviewed and approved by the Research Ethics Committee of the Hospital de Reabilitação de Anomalias Craniofaciais, Universidade de São Paulo (protocol 4.658.281) and registered in the Brazilian Registry of Clinical Trials (ReBEC; protocol RBR-8x8pf6d). The study evaluated dental models, which are part of patient documentation at the rehabilitation center.

### Sample

The sample consisted of participants with non-syndromic unilateral cleft lip and palate. All surgical procedures were performed by a single plastic surgeon at the same rehabilitation center. Participants with other associated anatomical-functional anomalies and those who did not have dental models by the institution's rehabilitation protocol and/or were of inadequate quality were excluded from the selection.

**Figure 1:**
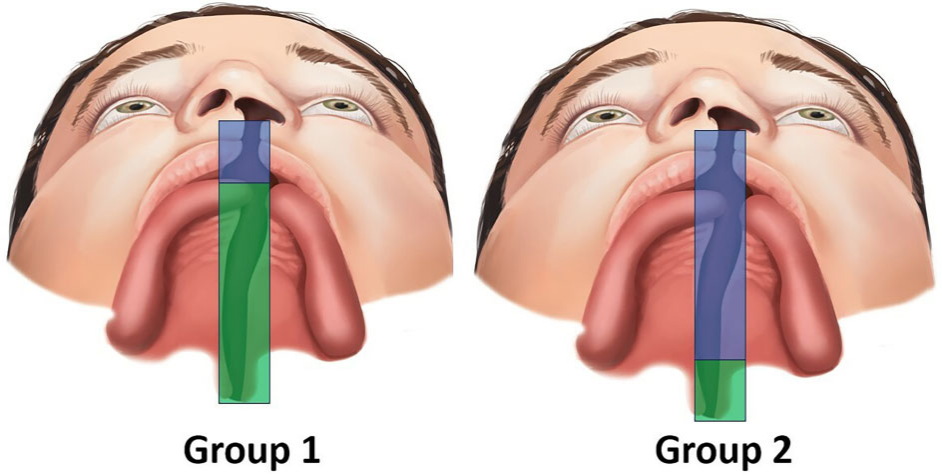
Group 1: Millard technique (cheiloplasty) at three months of age (blue) and von Langenbeck technique (one-stage palatoplasty) at 12 months (green). Group 2: Millard technique and Hans Pichler technique (hard palate repair) at three months (blue); Sommerlad technique (soft palate repair) at 12 months (green).

**Figure 2:**
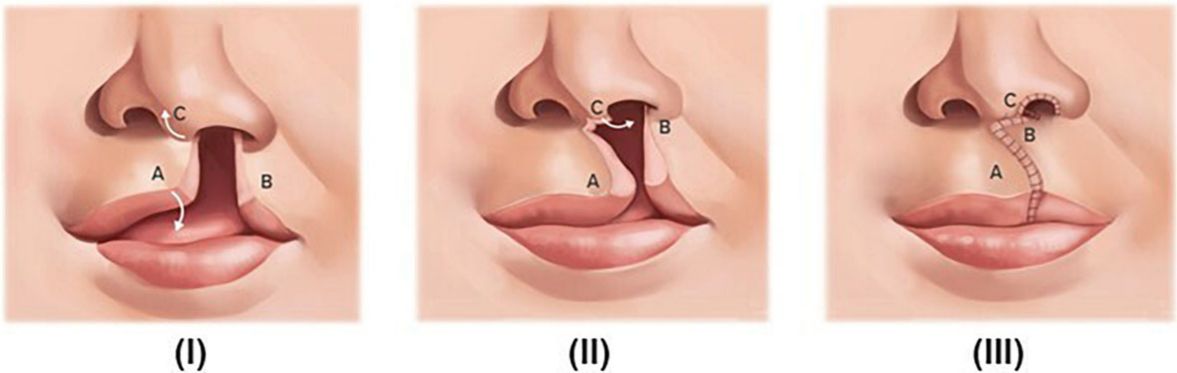
Millard Technique (cheiloplasty). Surgical flaps were made on the side without cleft for inferior rotation of the medial lip element (A); the medial advancement flap - side with cleft (B); and the base flap on the columella (C). **I**, Preoperative view; **II**, Transoperative view; **III**, Postoperative view.

**Figure 3:**
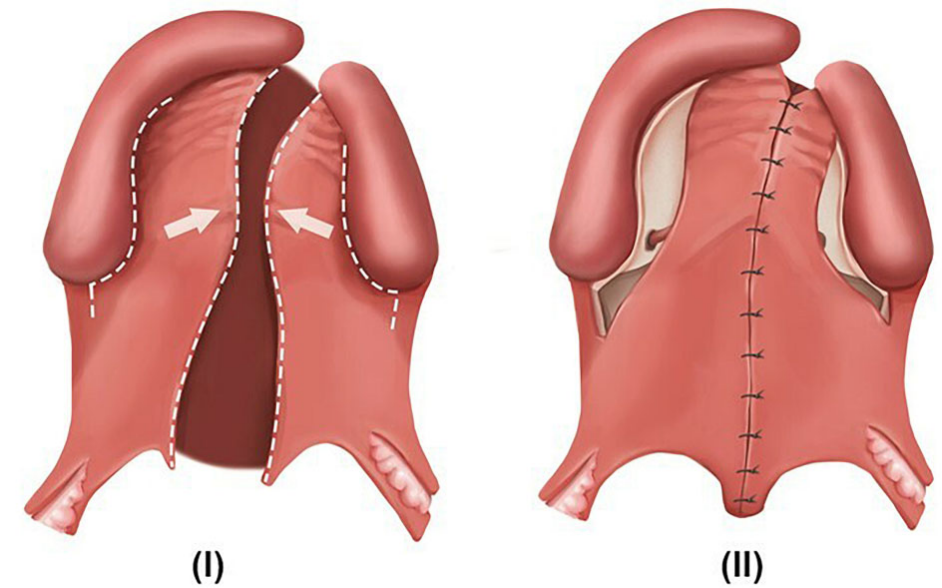
von Langenbeck technique (palatoplasty). **I**, Preoperative view: incisions were made on the margin of the cleft (from the alveolar region to the uvula), and relaxing incisions were made from the canine to the retromolar region. The incisions were made on the sides with and without cleft. **II**, Postoperative view.

G*Power software (version 3.1.9.7; Heinrich-Heine-Universität Düsseldorf, Germany) was used for sample size calculation with α=5% and β=20%. In addition, a d-value of 0.86 (effect size) was considered for the parameter of cleft depth in the smaller segment, according to a previous study. The analysis indicated that the present study should have at least 23 participants/group. Thus, 28 individuals were allocated into each of two groups (Figure 1) according to the different surgical techniques. Group 1 (G1) included participants who had undergone the Millard technique (Figure 2) for cheiloplasty at three months of age and the von Langenbeck one-stage palatoplasty (Figure 3) at 12 months. Group 2 (G2) included participants who had undergone the Millard technique (Figure 2) for cheiloplasty at three months of age and simultaneous repair of the hard palate using the Hans Pichler technique; at 12 months of age, soft palate palatoplasty was performed using the Sommerlad technique. All participants had their palate molded at the pediatric dentistry clinic at the following stages: preoperative 1 (T1, at three months of age), preoperative 2 (T2, at 12 months of age), and postoperative (T3, at 24 months of age).

### Procedures

All impressions were sent to the dental laboratory of the rehabilitation center to obtain plaster dental models. After this stage, the models were scanned by a 3D scanner (R700™, 3Shape, Denmark) with proven accuracy in previous studies. The virtual models were exported in Standard Tesselation Language (STL) format and subsequently analyzed using Mirror¯ software (Canfield Scientific, USA) (15).

### Digital morphometric analyses

**Figure 4:**
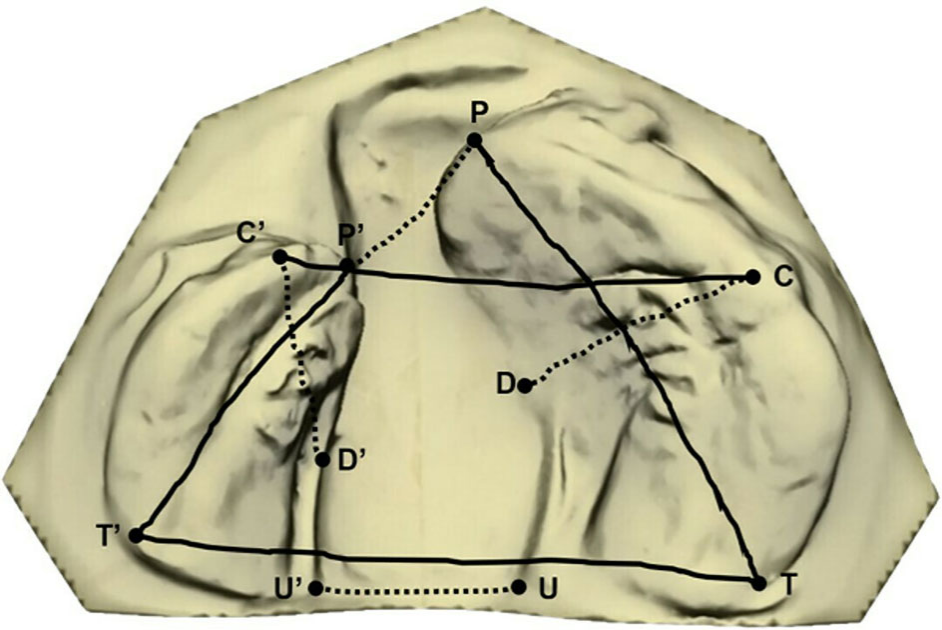
Across-surface distances evaluated in dental arches. Anterior (P-P′) and posterior (U-U′) cleft widths, intercanine (C-C′) and intertuberosity (T-T′) widths, lengths of the smaller (P′-T′) and larger (P-T) segments, and depths of the cleft in the smaller (C′-D′) and larger (C-D) segments.

The software manually marked anatomical points according to published articles (14,16). Among these points, across-surface distances anterior (P-P′) and posterior (U-U′) cleft widths, intercanine (C-C′) and intertuberosity (T-T′) widths, lengths of the smallest (P′-T′) and largest (P-T) segments, and cleft depths in the smallest (C′-D′) and largest (C-D) segments were evaluated (16) (Figure 4). All analyses were automatically calculated by the software and quantified in mm.

### Percent growth rate (GR)

The palatal growth was quantified in percentage and evaluated for all distances measured, according to the following formula: GR = (parameter T3 - parameter T1) × 100 / Parameter T1, where T1 = preoperative time 1 and T3 = postoperative time.

### Statistical analysis

All across-surface distances were evaluated by Jamovi (computer software, version 2.3, Australia) and tested for normality (Shapiro-Wilk test). Fifty-six dental models (1/3 of the sample) were randomly selected for intra-rater (paired *t*-test) and inter-rater (Mann-Whitney test) agreement analysis. All parameters were quantified twice at an interval of 15 days (17). Paired *t*-test, Wilcoxon test, repeated measures ANOVA/Tukey test, and Friedman test/Dunn's multiple comparison test were used to compare within-group differences. Independent *t*-test and Mann-Whitney test were used to compare between-group percentage growth rates. Simple linear regression analysis and F-test were also applied. Descriptive data are reported as means±SD and median±interquartile range.

## Results

### Sample

One hundred and sixty-eight dental models were digitally evaluated, 84 from group 1, referring to 28 participants, and 84 from group 2 (28 participants). The impressions were taken at three different times, with the following mean ages in years: 0.35 (T1), 1.17 (T2), and 2.16 (T3).

### Intra- and inter-rater reliability

There was no systematic error between the evaluated intra-rater (P=0.556, paired *t*-test) and inter-rater (P=0.491, Mann-Whitney test) distances, indicating that the measurements were reproducible.

### Intragroup comparisons

In group 1, P-P′, U-U′, and C-C′ showed significant reductions after cheiloplasty (T2), unlike P′-T′, which increased (P<0.001, P=0.030, P<0.001, and P<0.001, respectively). Furthermore, the P-T and C′-D′ distances showed significant growth only after one-stage palatoplasty (T3) (P<0.001, P=0.003, in this order), while C-D increased at all stages (P<0.001) (Table 1).

**Table 1:** Analysis between time points 1 (preoperative 1), 2 (preoperative 2), and 3 (postoperative) of the group treated with cheiloplasty and one-stage palatoplasty (G1).

Across surface distance	T1	T2	T3	P-value
P-P′	19.21±8.61	4.37±4.54	-	<0.001^#^
U-U′	17.16±4.31	14.78±4.15	-	0.030
C-C′	43.87±7.16^A^	29.99±6.94^B^	29.72±4.92^B^	<0.001^#^
T-T′	40.61±6.63	40.98±6.53	38.97±3.72	0.113^#^
P′-T′	24.1±1.42^A^	27.55±2.54^B^	28.4±2.61^B^	<0.001
P-T	34.99±2.71^A^	36.21±3.1^A^	37.84±3.72^B^	<0.001
C′-D′	17.09±1.74^A^	16.84±1.53^A^	18.66±2.88^B^	0.003
C-D	19.29±1.27^A^	21.26±1.69^B^	23.7±2.59^C^	<0.001^#^

Paired *t*-test and repeated measures ANOVA followed by the Tukey test (data reported as mean±standard deviation). ^#^Wilcoxon test and Friedman test followed by Dunn's multiple comparison test (data reported as median±interquartile range). Different superscript capital letters indicate a statistically significant difference between times. Anterior (P-P′) and posterior (U-U′) cleft widths, intercanine (C-C′) and intertuberosity (T-T′) widths, lengths of the smaller (P′-T′) and larger (P-T) segments, and depths of the cleft in the smaller (C′-D′) and larger (C-D) segments.

In group 2, U-U′ and C-C′ showed significant reduction after cheiloplasty (T2) (P=0.001 and P<0.001, respectively). On the other hand, the distances P′-T′, P-T, C′-D′, and C-D showed significant growth after two-stage palatoplasty (T3) (P<0.001, for all) (Table 2).

**Table 2:** Analysis between time points 1 (preoperative 1), 2 (preoperative 2) and 3 (postoperative) of the group treated with cheiloplasty and two-stage palatoplasty (G2).

Across surface distance	T1	T2	T3	P-value
U-U′	16.85±3.86	12.22±3.12	-	0.001
C-C′	40.83±7.46^A^	30±3.58^B^	32.22±3.69^B^	<0.001
T-T′	42.79±4.49	42.32±3	42.48±4.35	0.893
P′-T′	24.42±3.31^A^	27.28±4.1^A^	30.13±4.75^B^	<0.001^#^
P-T	34.67±3.55^A^	36.07±3.28^A^	37.88±4.5^B^	<0.001^#^
C′-D′	16.97±2.13^A^	15.85±2.34^A^	20.37±2.99^B^	<0.001
C-D	18.94±1.99^A^	19.66±5.04^A^	24.78±4.17^B^	<0.001^#^

Paired t-test and repeated measures ANOVA followed by the Tukey test (data reported as mean±standard deviation). ^#^Friedman test followed by Dunn′s multiple comparison test (data reported as median± interquartile range). Different superscript capital letters represent a statistically significant difference between times. Posterior (U-U′) cleft width, intercanine (C-C′) and intertuberosity (T-T′) widths, lengths of the smaller (P′-T′) and larger (P-T) segments, and depths of the cleft in the smaller (C′-D′) and larger (C-D) segments.

### Intergroup comparisons

There was no statistically significant difference in the comparison between groups at time 1 (P-P′, P=0.169; U-U′, P=0.782; C-C′, P=0.132; T-T′, P=0.281; C′-D′, P=0.826, independent *t*-test and P′-T′, P=0.248; P-T, P=0.571; C-D, P=0.187, Mann-Whitney test).

In general, the growth rate in group 2 was higher compared to group 1; however, only C′-D′ was significant (P=0.038) (Table 3).

**Table 3:** Comparison of percentage growth rate between the group treated with cheiloplasty and one-stage palatoplasty (G1) and the group treated with cheiloplasty and two-stage palatoplasty (G2).

Across surface distance	G1	G2	P-value
C-C′	-23.26±18.51	-18.91±15.36	0.343
T-T′	-2.94±17.74	-1.81±17.92	0.274^#^
P′-T′	18.16±11.99	23.61±22.51	0.263
P-T	6.98±13.36	6.55±16.18	0.700^#^
C′-D′	10.06±3.55	21.41±4	0.038
C-D	21.15±16.27	28.88±29.29	0.103^#^

Independent *t*-test (data reported as mean±standard deviation). ^#^Mann-Whitney test (data reported as median ± interquartile range). Growth rate = (parameter T3 - parameter T1) × 100 / Parameter T1. Intercanine (C-C′) and intertuberosity (T-T′) widths, lengths of the smaller (P′-T′) and larger (P-T) segments, and depths of the cleft in the smaller (C′-D′) and larger (C-D) segments.

A simple linear regression analysis was performed in which the rehabilitation protocol was the independent variable and the measurements that presented normal distribution (T-T′ and C′-D′) were the dependent variables. The C′-D′ parameter showed a significant coefficient of determination (R^2^) of 0.076 (P=0.039), which means that the rehabilitation protocol explained 7.6% of the results of this distance. In other words, this model indicated that the rehabilitation protocol influenced the outcome (Table 4).

**Table 4:** Simple linear regression between rehabilitation protocol (independent variable) and growth rate (dependent variable).

Model	R	R^2^	Equation	SEiv	SEdv	F-change	P-value
T-T′	0.149	0.022	y=0.17-4.01x	2.57	3.63	1.22	0.274
C′-D′	0.277	0.076	y=21.4-11.4x	3.79	5.36	4.49	0.039

F-test. R: Pearson correlation coefficient. R^2^: Coefficient of determination. Seiv: Standard error of independent variable. Sedv: Standard error of dependent variable. Intertuberosity width (T-T′); depths of the cleft in the smaller segments (C′-D′).

## Discussion

In this study, one- and two-stage palatoplasty were evaluated in participants with unilateral cleft lip and palate operated on by a single plastic surgeon. Comparisons were made using dental models scanned with the across-surface distance method. This tool measures the shortest surface distance along a path defined by two anatomical points (16). Unfortunately, this tool needs to be used more in the analysis of dental arch development (16) and the face (18,19) of individuals with an orofacial cleft as more software tools make this anthropometric analysis tool available. However, its applicability should be disseminated to complement the knowledge of researchers and professionals who rehabilitate children with an orofacial cleft.

In group 1, both the intercanine measurement (C-C′) and the anterior (P-P′) and posterior (U-U′) cleft widths decreased after cheiloplasty (T2), indicating approximation between the alveolar segments in both the anterior and posterior portions of the dental arch. Although other authors have performed these analyses using the straight-line method, the data from this study corroborate the previous findings (11,12,20). In contrast, the lengths of the palatal segments, P′-T′ and P-T, and the cleft depths, C′-D′ and C-D, grew significantly from T1 to T3. Previous authors also reported total palate growth, measured as the distance from the inter-incisive point to the intertuberosity distance, after palatoplasty (13,21).

In group 2, the C-C′ distance and the posterior cleft width (U-U′) reduced after the first surgical step, i.e. cheiloplasty with anterior palatoplasty (T2). As in G1, group 2 also showed significant growth in palatal segment lengths (P′-T′ and P-T) and cleft depths (C′-D′ and C-D) between T1 and T3. These findings corroborate other data published in the scientific literature, in which the authors evaluated children with the same orofacial cleft phenotype but received different rehabilitation protocols (16).

Comparing the groups at T1, there was no statistically significant difference in the analyses before the surgical procedures. This finding is important, indicating no baseline discrepancies between groups. However, at T2, the growth rate of group 2 had higher growth percentages in five (C-C′, T-T′, P′-T′, C′-D′, and C-D) of the six parameters evaluated, but only C′-D′ showed a statistically significant difference. These data suggested that individuals who underwent two-stage palatal surgical repair showed better palatal growth, consistent with previous studies (22-25). One systematic review on the effect of one- or two-stage palatoplasty on maxillofacial development was inconclusive due to contradictory results. However, the authors reinforce that the different surgical protocols and the age of the participants at assessment may have interfered with the inconclusive results (8).

It is well established in the literature that the development of dental arches of children with oral cleft can be influenced by various factors, such as genetics (9), treatment protocols (16), surgical techniques (17), age at surgeries (26), width of the cleft (9), in addition to the intrinsic characteristics of individuals. Although several studies (13,27,28) reinforce that the rehabilitation protocol influences palatal development, specific statistical analyses are not always applied to confirm this concept. This study used a simple linear regression analysis to evaluate the influence of the rehabilitation protocol on the quantitative parameters evaluated. As this analysis is conditioned to normally distributed data, only the T-T′ and C′-D′ parameters were assessed. The T-T′ distance was not significantly influenced by the rehabilitation protocol, but for the C′-D′ distance, the protocol explained 7.6% of the variation and this was statistically significant. These data reinforce that, in the evaluated model, the rehabilitation protocol affected palatal growth, especially the C′-D′ distance, corroborating the data from the between-group comparison of percentage growth rates.

The highlight of this study was that a single plastic surgeon operated on all participants; the involvement of more than one surgeon could bias the results (13). However, the existing limitation was the lack of classification for cleft width, i.e. wide, medium, or narrow. Therefore, this parameter could not be evaluated in the sample. Studies must be designed considering this vital factor, as wide clefts are challenging for surgeons and tend to lead to greater limitations in palatal development (9,10,14). Another point that should be considered is that, due to the absence of studies that evaluate the influence of the rehabilitation protocol from childhood to skeletal maturity, it is essential to perform comparative studies with other rehabilitation centers that have different rehabilitation protocols.

The evidence from this study allows us to conclude that, regardless of the surgical technique, dental arch development is influenced by the rehabilitation protocol. In the sample evaluated, individuals with a cleft palate operated on in two stages showed the most favorable palatal growth.
